# Comprehensive evaluation of an emergency monovalent SAT1 foot-and-mouth-disease vaccine: Antigen quality, product consistency, and protective efficacy

**DOI:** 10.1371/journal.pone.0353701

**Published:** 2026-07-15

**Authors:** Mohamed Samy Abousenna, Nermeen G. Shafik, Sara E.A. El Sawy, Amal Abd El Moneim Mohamed, Heba A. Khafagy, Ehab El-Sayed Ibrahim, Wael Elfeil, Mohamed Ahmed Saad, Samir A. Nassif

**Affiliations:** 1 Agricultural Research Center, Central Laboratory for Evaluation of Veterinary Biologics, Cairo, Egypt; 2 Agricultural Research Center, Veterinary Serum and Vaccine Research Institute, Cairo, Egypt; 3 Avian and Rabbit Medicine Department, Faculty of Veterinary Medicine, Suez Canal University, Ismailia, Egypt; Sudan University of Science and Technology, College of Veterinary Medicine, SUDAN

## Abstract

Foot-and-mouth disease (FMD) remains endemic in Egypt, with periodic incursions of antigenically distinct virus lineages. In 2025, FMDV serotype SAT1/topotype I was detected in Egypt for the first time, creating an immunity gap in a livestock population previously vaccinated against serotypes O, A, and SAT2. This study aimed to evaluate the quality, immunogenicity, antigenic relationships, and protective efficacy of an emergency inactivated monovalent SAT1 vaccine and to assess potential cross-reactivity with SAT2 vaccine strains. An inactivated SAT1 monovalent vaccine was produced under emergency conditions and subjected to in-process and final product quality control testing, including sterility, safety, RT-qPCR verification, and 146S antigen quantification. Neutralizing antibody responses were measured by virus neutralization test (VNT) at 28 days post-vaccination. Antigenic relationships between SAT1 and SAT2 strains were assessed using r₁-value analysis. Protective efficacy was evaluated by homologous challenge in vaccinated calves. All vaccine batches met quality control criteria, with consistent viral titers and 146S antigen content. Vaccinated calves developed a mean homologous VNT titer of 2.175 log₁₀ TCID₅₀, exceeding the WOAH protective threshold (≥1.65 log₁₀ TCID₅₀). Following homologous challenge, 100% (8/8) of vaccinated calves were clinically protected. In contrast, heterologous VNT titers against SAT2 strains were low (0.26–0.30 log₁₀ TCID₅₀), and r₁-values ranged from 0.12 to 0.14, below the WOAH threshold for antigenic matching (0.3). The emergency SAT1 monovalent vaccine induced strong homologous immunity and complete protection against SAT1 challenge. Limited antigenic and serological cross-reactivity with SAT2 confirms the serotype-specific nature of FMD immunity and supports the need for dedicated SAT1 vaccination and ongoing antigenic surveillance.

## Introduction

Foot-and-mouth disease (FMD) is a highly contagious transboundary viral infection of cloven-hoofed animals and remains one of the most economically devastating diseases affecting global livestock production [1]. It impacts a broad range of species—including cattle, pigs, sheep, and goats—and is clinically characterized by fever and the development of vesicular lesions on the oral mucosa, tongue, feet, and teats, often resulting in lameness, reduced feed intake, and marked productivity losses [2]. FMD virus (FMDV) is a small, non-enveloped, positive-sense RNA virus of approximately 8.5 kb, classified within the genus *Aphthovirus* (family *Picornaviridae*) [3]. Its considerable antigenic and genetic diversity is reflected in seven immunologically distinct serotypes—O, A, C, Asia 1, SAT1, SAT2, and SAT3—none of which confer cross-protection [4]. Several of these serotypes continue to circulate periodically in the Middle East and North Africa, making continuous adaptation of vaccination strategies essential for effective disease control [5]. The global epidemiological complexity of FMD underscores the need for serotype-specific vaccines, robust surveillance, and timely antigenic updates to sustain protection and limit transboundary spread.

The virus spreads through direct or indirect contact with infected animals, contaminated fomites, animal products, and aerosols capable of long-distance transmission [6]. Additional risk factors include animal movement, human mobility, and interactions between domestic and wildlife populations [7]. Viral detection in milk and semen often precedes the onset of fever and vesicular lesions on the oral mucosa, teats, and interdigital spaces, complicating early diagnosis [8]. While mortality in adult animals is generally low, young animals are highly susceptible to fatal myocarditis, and recovered animals may become long-term carriers capable of reintroducing infection [9].

FMD has been endemic in Egypt since the 1950s, with repeated incursions of serotypes O, A, and SAT2 [10]. Serotype O has remained the predominant strain and forms the antigenic foundation of Egypt’s routine vaccine formulations [11]. Significant antigenic and lineage shifts have occurred over the decades. In 2006, Egypt recorded the introduction of an East African A strain, representing the first confirmed long-distance incursion from Pool 4 [12]. Between 2010 and 2015, multiple outbreaks associated with the A-Iran05–08 lineage were reported, indicating continued viral introductions from the Middle East [9]. From 2012 onward, the A/Africa G-IV topotype has persisted, showing approximately 93.7% VP1 nucleotide similarity among circulating strains but accumulating amino-acid substitutions at key antigenic sites, suggesting ongoing antigenic drift [11,12].

Parallel to these developments, the O ME-SA PanAsia-2 lineage, once dominant in Egypt, was replaced during 2014–2016 by the O/EA-3 lineage, prompting reassessment of serotype O vaccine components [13]. SAT2, first detected in 2012, re-emerged forcefully in 2018 as the Lib-12 lineage (topotype VII), causing widespread disease despite high vaccination coverage [6]. Phylogenetic analyses linked these SAT2 viruses to sub-Saharan lineages, revealing Egypt’s vulnerability to external viral introductions through trade and regional livestock movement [14].

Recent years have seen the emergence of genetically and antigenically divergent lineages. The FMDV-A-Egy-AHRI-RL385-Ven-2022 strain demonstrated an unexpected phylogenetic relationship to the EURO-SA lineage and showed antigenic overlap with A/Africa G-IV, underscoring the co-circulation of heterogeneous A lineages in Egypt [11,12]. Critically, in July–August 2025, Egypt confirmed its first incursion of SAT1 topotype I in Al Buhayrah Governorate [15]. Confirmation by the World Organisation for Animal Health (WOAH) and the World Reference Laboratory for FMD (WRLFMD) demonstrated that animals vaccinated with conventional trivalent (O, A, SAT2) vaccines lacked SAT1-specific antibodies, revealing an immunity gap and contributing to rapid spread in small ruminants [16,17].

The emergence of FMDV SAT1 in Egypt represented a significant epidemiological event because SAT1 had not been previously established among the circulating serotypes affecting Egyptian livestock. The introduction of a novel SAT1 lineage increased the risk of rapid disease spread within susceptible cattle populations and raised concerns regarding the adequacy of existing vaccine formulations. Consequently, the development and evaluation of a SAT1-specific emergency vaccine became a national priority to support outbreak preparedness and vaccine-matching activities [18].

Vaccine performance and antigenic fit remain central to FMD control. Over the past decade, extensive evaluations conducted by the Central Laboratory for Evaluation of Veterinary Biologics (CLEVB) have shown that antigenic drift, particularly within serotypes A and SAT2, reduces cross-protection provided by existing antigens [11,12]. r₁-values below 0.3 have been reported for several newly emerged lineages—including A EURO-SA, A Africa G-IV, and SAT2 Lib-12—indicating weak antigenic relatedness and increased risk of vaccine failure [6]. Conversely, studies have demonstrated that strategic inclusion of antigenically representative strains within vaccine formulations, such as O PanAsia-2, can sustain broad neutralization against divergent field strains when critical epitopes remain conserved [13,19].

As a result of these findings, updated polyvalent vaccines in Egypt now incorporate A EURO-SA, A Africa G-IV, and SAT2 Lib-12. However, the emergence of SAT1 topotype I presents a new and urgent challenge, as this serotype has not previously circulated in Egypt and remains absent from existing polyvalent vaccines [16,19]. In response, the Veterinary Serum and Vaccine Research Institute (VSVRI) developed an emergency monovalent SAT1 vaccine for rapid deployment in affected areas, with plans for integration into future polyvalent formulations pending expanded matching and surveillance data.

Accordingly, this study was designed as a preliminary, multi-parameter evaluation of a newly developed monovalent SAT1 foot-and-mouth disease vaccine in response to the first confirmed SAT1 incursion in Egypt. In accordance with WOAH guidelines for FMD vaccine assessment, the study integrates key components of vaccine evaluation, including antigen quality control, manufacturing consistency, antigenic characterization, immunogenicity assessment, in vitro cross-neutralization, and in vivo protection. Beyond the expected protective outcome of a homologous SAT1 vaccine, this framework links vaccine production quality parameters directly with functional immune responses under outbreak conditions. Unlike previous FMD vaccine studies that typically evaluate immunogenicity, antigenic matching, or protection in isolation, this work provides a continuous quality-to-immunity evaluation pipeline. This study therefore generates early, complementary evidence to support emergency vaccine deployment and refinement, rather than serving as a definitive assessment of long-term field effectiveness or antigenic coverage breadth.

## Materials and methods

**Fig 1:**
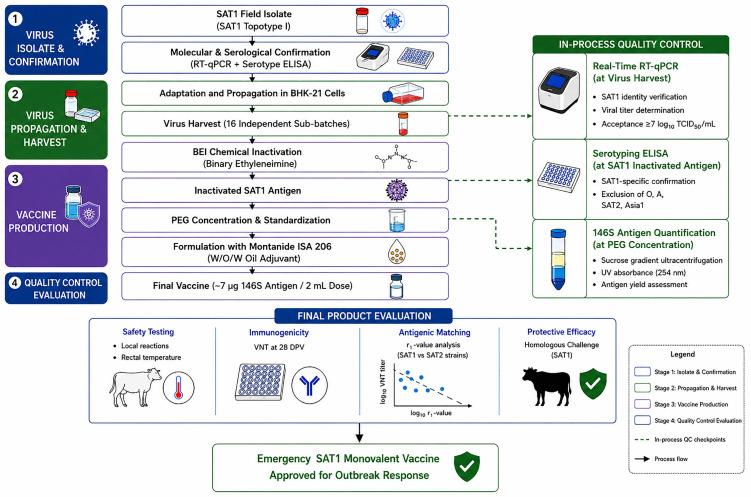
Overview of SAT1 vaccine production, in-process quality control, and final product evaluation. The schematic illustrates the workflow used for the development and evaluation of an emergency SAT1 monovalent foot-and-mouth disease (FMD) vaccine. The process included SAT1 field virus isolation and confirmation, adaptation and propagation in BHK-21 cells, virus harvest, chemical inactivation using binary ethyleneimine (BEI), antigen concentration and standardization, formulation with Montanide ISA 206 adjuvant, and final vaccine preparation. In-process quality control procedures comprised real-time RT-qPCR of harvested virus, serotyping ELISA of the inactivated SAT1 antigen, and 146S antigen quantification following PEG concentration. Final product evaluation included safety testing, immunogenicity assessment by virus neutralization test (VNT), antigenic matching analysis (r₁-value determination), and protective efficacy evaluation following homologous SAT1 challenge. **Abbreviations:** BEI, binary ethyleneimine; ELISA, enzyme-linked immunosorbent assay; FMD, foot-and-mouth disease; RT-qPCR, real-time reverse transcription polymerase chain reaction; SAT1, Southern African Territories serotype 1; VNT, virus neutralization test.

A schematic overview of the SAT1 vaccine production process, in-process quality control procedures, and final product evaluation is presented in Fig 1**.**

### In-process evaluation of the inactivated SAT1 monovalent FMD vaccine

#### Real-time RT-PCR verification of FMDV SAT1 during in-process vaccine production.

Samples were collected from the Master Seed Foot-and-Mouth Disease Virus SAT1 (FMDV SAT1) cultures during in-process quality control of the monovalent vaccine manufacturing line. Following propagation of the SAT1 Master Seed virus in BHK-21 cell monolayers, aliquots were taken from 16 independent sub-batches prior to chemical inactivation and antigen formulation. These samples were tested to verify the presence and genetic identity of FMDV SAT1 before continuing downstream vaccine production.

Real-time RT-PCR assays were performed using the Foot-and-Mouth Disease Virus SAT1 (FMDV-SAT1) Probe qRT-PCR Kit (Biofargo Inc., Beijing, China, Catalog No. 15–17750) according to the manufacturer’s instructions. The kit employs a one-tube hydrolysis-probe system in which viral RNA is reverse-transcribed and amplified using SAT1-specific primers and a FAM-labeled probe targeting a conserved SAT1 region of the VP1 gene.

**RNA Extraction:** Viral RNA was extracted from each sub-batch sample using the QIAamp Viral RNA Mini Kit (Qiagen, Hilden, Germany). Purification Positive Control (PPC; known SAT1-positive sample) and Purification Negative Control (PNC; known negative sample) were included with each extraction batch to ensure RNA integrity and absence of contamination.

**Reaction Preparation:** For each reaction, 14 µL of PCR premix (Probe qRT-PCR Buffer, Enzyme Mix, and reconstituted SAT1 Primer-Probe Mix) was combined with 6 µL of extracted RNA. An Amplification Positive Control (APC; kit-provided SAT1 cDNA at 10⁷ copies/µL) and an Amplification Negative Control (ANC; RNase-free water) were included in each run. The lyophilized SAT1 primer–probe mix was reconstituted once using RNase-free water per the user manual.

**Thermal Cycling Conditions:** Amplification was carried out using a CFX96 Real-Time PCR Detection System (Bio-Rad Laboratories, Hercules, CA, USA) under the recommended cycling profile: reverse transcription at 50 °C for 10 min; initial denaturation at 95 °C for 5 min; followed by 45 cycles of 95 °C for 15 s and 60 °C for 30 s, with fluorescence acquisition in the FAM channel.

#### Standard curve and quantification.

A seven-point standard curve was prepared by generating serial 10-fold dilutions of the APC covering the range 10¹ to 10⁷ copies/µL, following the protocol described in the kit manual. The regression equation for the curve was: y*=−0.2953x + 12.24* with a correlation coefficient: 𝑅^2^ = 0.9967.

The standard curve is shown in S1 Fig. Ct values obtained from the 16 sub-batches were converted to viral RNA quantities using the regression model derived from this standard curve.

**Interpretation:** A sub-batch was considered positive for FMDV SAT1 when a characteristic amplification curve with Ct < 40 was obtained, provided that APC amplified correctly and ANC remained negative. PPC and PNC performance was required to validate RNA extraction quality. Only sub-batches that passed all quality-control criteria were authorized to proceed to the inactivation and antigen preparation steps.

#### Identification of the prepared inactivated antigen using fmdv serotyping ELISA.

Serotype confirmation of the 16 inactivated SAT1 antigen sub-batches was performed using the FMDV Serotyping ELISA Kit (FMDV O, A, SAT1, SAT2, Asia 1; IZSLER, Brescia, Italy & The Pirbright Institute, UK; Lot No. 01-2019190301a). This assay was used to verify that each sub-batch retained its SAT1 identity following chemical inactivation and to exclude any contamination or unintended mixing with other FMD serotypes before the emulsion and formulation stages [11].

The ELISA was conducted strictly according to the manufacturer’s instructions provided in the kit insert. For each sub-batch, antigen was tested in parallel against all five serotype-specific monoclonal antibody–coated wells. A sub-batch was considered correctly identified as SAT1 when: A strong positive reaction was detected exclusively in the SAT1-specific wells, and no detectable reactivity was observed in the wells corresponding to serotypes O, A, SAT2, or Asia 1.

Only sub-batches confirmed as SAT1-positive and free from cross-serotype reactivity were approved to proceed to the vaccine emulsification and formulation steps.

#### Quantification of 146S antigen content in in-process SAT1 sub-batches.

Quantification of the intact 146S virion particles was performed for all 16 SAT1 in-process antigen sub-batches to ensure consistency of antigen yield prior to formulation of the monovalent FMD vaccine. This step serves as a critical quality-control measure, allowing adjustment and optimization of the final dose based on the actual concentration of immunogenic 146S particles. For each sub-batch, 0.2 mL of the extracted, clarified SAT1 antigen (post-inactivation and pre-formulation) was layered onto a 10–25% sucrose (Sigma-Aldrich, St. Louis, MO, USA) density gradient prepared in phosphate-buffered saline (PBS; Gibco, Thermo Fisher Scientific, Waltham, MA, USA). The gradient was constructed in 5 mL ultracentrifuge tubes by gently adding: 2.2 mL of 25% sucrose solution, followed by 2.2 mL of 10% sucrose solution, applied beneath the upper layer using a long syringe needle to maintain a stable interface [20].

Each antigen sample was carefully pipetted on top of the gradient. Tubes were centrifuged in a Kontron Centrikon T-1080 ultracentrifuge fitted with a swinging-bucket rotor at 45,000 rpm for 40 minutes at 4°C.

Following centrifugation, gradients were analyzed using an ISCO 520C density-gradient fractionation and UV monitoring system (Teledyne ISCO, Lincoln, NE, USA). Absorbance was continuously recorded at 254 nm, and the 146S antigen peak was identified according to its characteristic sedimentation position within the sucrose gradient. Peak areas were integrated and converted to 146S antigen concentrations using the conversion coefficient was derived from calibration experiments using purified FMDV 146S particles as described by Barteling and Meloen [20]. and subsequently adopted in FMD vaccine antigen quantification studies [11]:

*146S concentration (μg/mL) = Peak area (mm²) × 0.0116*

This analysis yielded the antigen concentration for each sub-batch, allowing verification of batch-to-batch uniformity and enabling adjustment of the final antigenic load to meet required potency specifications for the SAT1 monovalent vaccine [11,21].

### Final product evaluation of the inactivated SAT1 monovalent FMD vaccine

#### Virus and cells.

An Egyptian Foot-and-Mouth Disease Virus (FMDV) serotype SAT1 field isolate, phylogenetically classified within the SAT1/topotype I (North-West Zimbabwe) lineage, was initially detected at the Animal Health Research Institute (AHRI) using reverse transcription real-time PCR (RT-qPCR) combined with serotype-specific ELISA [15]. Following molecular and serological confirmation, the isolate was transferred to the Central Laboratory for Evaluation of Veterinary Biologics (CLEVB), Agricultural Research Center (ARC), for further antigenic and vaccine evaluation.

For molecular characterization, the VP1-encoding gene sequences of five SAT1 isolates obtained in this study were generated and deposited in the GenBank database under accession numbers PX252048, PX252049, PX252050, PX252051, and PX252052. Sequence analysis and BLAST comparison confirmed clustering within the SAT1/I topotype, consistent with the recently reported SAT1 incursion in Egypt [15], and supported molecular confirmation of the vaccine challenge strain used in this study.

The SAT1 isolate was subsequently adapted to baby hamster kidney (BHK-21) cell monolayers, resulting in a reproducible infectious titer of 10⁷ TCID₅₀/mL. The cell-adapted virus was employed as the homologous reference strain in virus neutralization tests (VNT). In parallel, the virulent SAT1 virus was titrated and used as the challenge strain in in vivo protection studies conducted in vaccinated calves, in accordance with WOAH-recommended procedures [21].

For assessment of antigenic relationships and heterologous neutralizing activity, two SAT2 strains—SAT2/EGY-2012 and SAT2/VII (Lib-12)—were obtained from the Strain Bank Department of CLEVB. These strains were selected based on their epidemiological relevance and were used in cross-neutralization assays to evaluate potential antigenic relatedness and cross-protective immune responses relative to the SAT1 vaccine strain, following WOAH guidelines [21].

Baby hamster kidney (BHK-21) cells, a continuous cell line extensively validated for Foot-and-Mouth Disease Virus propagation and serological applications, were used throughout virus amplification, virus neutralization testing, and antigenic relationship analyses. The cells were supplied by the Foot-and-Mouth Disease Vaccine Production Department of the Veterinary Serum and Vaccine Research Institute (VSVRI), Abbassia, Cairo, Egypt.

### Vaccine production, inactivation, and formulation

Virus propagation was performed in BHK-21 cell monolayers under GMP-compliant conditions at the Veterinary Serum and Vaccine Research Institute (VSVRI), Cairo, Egypt, using a seed-lot system consistent with WOAH recommendations for FMD vaccine production. Cells were infected with SAT1 virus and incubated until complete cytopathic effect was achieved.

Infected cell culture supernatants were harvested by decantation and clarified by low-speed centrifugation to remove cellular debris. The clarified viral suspension was concentrated using polyethylene glycol (PEG 6000) precipitation to enhance antigen yield prior to downstream processing, in accordance with established FMD vaccine manufacturing practices.

Virus inactivation was carried out using binary ethyleneimine (BEI) under validated conditions ensuring complete loss of infectivity while preserving 146S antigen integrity. Following inactivation, the antigen suspension was further clarified, standardized, and emulsified with Montanide ISA 206 oil adjuvant (Seppic, France) to produce a stable water-in-oil emulsion vaccine following WOAH-aligned inactivated FMD vaccine formulation procedures. Following emulsification with Montanide ISA 206 oil adjuvant, the final vaccine formulation was standardized to contain approximately 7 µg of 146S antigen per 2 mL field dose, in accordance with internal potency specifications for inactivated FMD vaccines used in national control programs.

#### Vaccine samples.

Recently manufactured batches of a monovalent inactivated SAT1 vaccine produced by the Veterinary Serum and Vaccine Research Institute (VSVRI, Cairo, Egypt) were obtained and evaluated at the Central Laboratory for Evaluation of Veterinary Biologics (CLEVB). Representative samples were selected to reflect the quality and consistency of the released production lot and to provide a reliable assessment of vaccine performance prior to deployment in the national vaccination campaign aimed at controlling the SAT1 incursion. The vaccine consisted of chemically inactivated FMDV SAT1 antigen formulated with an oil-based adjuvant. A single field dose was administered subcutaneously in the neck region using sterile disposable needles. Vaccine batches were maintained under refrigerated cold-chain conditions (2–8 °C) prior to administration in accordance with manufacturer recommendations. All animals enrolled in the study were clinically healthy, seronegative for FMDV antibodies, and had not received concomitant vaccines or medications during the experimental period.

#### Experimental design.

All vaccination and experimental procedures were implemented in accordance with the technical specifications provided by the manufacturer of the monovalent inactivated Foot-and-Mouth Disease Virus (FMDV) SAT1 vaccine.

A **Animals**

Twelve apparently healthy local Egyptian-breed male calves, aged between 6 and 8 months and weighing approximately 120–180 kg, were obtained from the Central Laboratory for Evaluation of Veterinary Biologics (CLEVB), Agricultural Research Center (ARC). All calves were clinically examined prior to the start of the experiment and confirmed to be free of detectable disease. Before enrollment, each animal was evaluated serologically for the presence of neutralizing antibodies against Foot-and-Mouth Disease Virus (FMDV) serotypes SAT1, SAT2/EGY-2012 (SAT2 GH), and SAT2/VII (Lib-12; SAT2 Libya) using the virus neutralization test (VNT). Only animals confirmed to be seronegative for all tested serotypes were included in the study. During the experimental period, animals did not receive any concomitant vaccines, medications, or immunomodulatory treatments [9,19].

Throughout the experimental period, calves were maintained in separate housing units within the CLEVB animal facility to prevent inadvertent exposure between groups. Animals were assigned to experimental categories based on their intended role in vaccination, challenge, or virus titration. Eight calves received a single subcutaneous field dose of a locally produced oil-adjuvanted inactivated SAT1 FMDV vaccine, while the remaining animals were designated for control and titration purposes.

**Fig 2:**
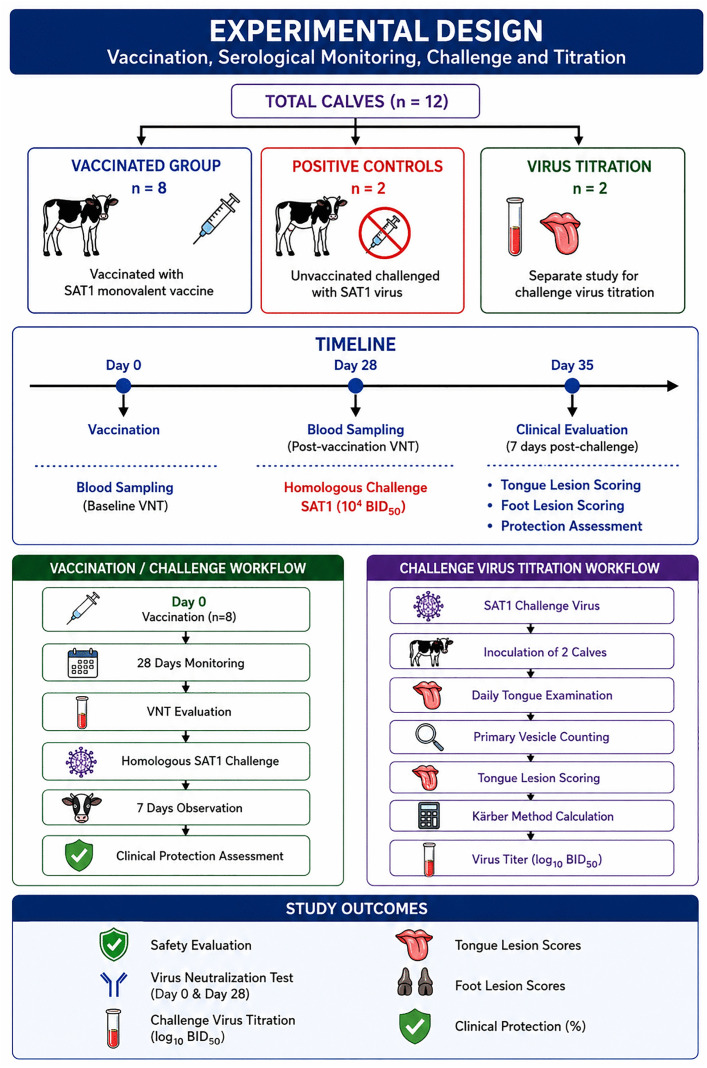
Experimental design of vaccination, serological monitoring, homologous challenge, and challenge virus titration. Schematic representation of the animal experiment conducted to evaluate the immunogenicity and protective efficacy of the SAT1 monovalent inactivated foot-and-mouth disease (FMD) vaccine. Twelve calves were allocated into three groups: a vaccinated group (n = 8), a positive control group (n = 2), and a separate virus titration group (n = 2). Calves in the vaccinated group received a single intramuscular dose of the SAT1 vaccine on Day 0 and were monitored for 28 days. Serum samples were collected on Days 0 and 28 for assessment of neutralizing antibody responses by virus neutralization test (VNT). On Day 28, vaccinated and control calves were challenged with a homologous SAT1 field isolate administered by the intradermolingual route. Animals were observed for 7 days post-challenge and evaluated for clinical protection based on tongue and foot lesion scores. Protection was determined by the absence of generalized lesions following challenge. The virus titration group was used independently for determination of challenge virus infectivity by inoculation of calves, daily tongue examination, lesion scoring, and calculation of virus titer using the Kärber method. Study outcomes included vaccine safety, neutralizing antibody responses, challenge virus titration, tongue and foot lesion scores, and clinical protection. Abbreviations: BID₅₀, 50% bovine infectious dose; FMD, foot-and-mouth disease; SAT1, Southern African Territories serotype 1; VNT, virus neutralization test.

The experimental design, animal grouping, vaccination schedule, post-vaccination monitoring, homologous challenge, and virus titration procedures are summarized in Fig 2

B **Experimental groups**

Group 1: Two calves used for in vivo titration of the SAT1 challenge virus.

Group 2: Eight vaccinated calves exposed to the homologous FMDV SAT1 strain.

Group 3 (Positive Control): Two unvaccinated calves challenged with the SAT1 strain.

All animal-related procedures were conducted following approval by the Institutional Animal Care and Use Committee (IACUC) of the Agricultural Research Center. Experimental practices adhered to nationally accepted ethical standards and were performed in line with previously established protocols described by Abousenna, et al. [9] and Nermeen, et al. [12].

#### Vaccine safety.

All vaccinated animals (Group 2) were closely monitored for clinical signs and any abnormalities. Injection sites were examined for adverse reactions, and any visible or palpable changes were documented in detail, as the study was conducted in an unblinded manner. Body temperature was recorded daily for 14 days post-vaccination, in addition to monitoring for 4 days prior to vaccination, and continued throughout the vaccine-efficacy study [19,21].

#### Virus neutralization test.

Post-vaccination blood samples were collected at 28 days from vaccinated group (Group 2) and analyzed using the virus neutralization test (VNT). The assay was performed on BHK-21 cells employing the microtiter neutralization technique as described by Ferriera and WOAH [21,22]. Cell cultures were maintained in Dulbecco’s Modified Eagle Medium (DMEM; Gibco, Thermo Fisher Scientific, Waltham, MA, USA) supplemented with fetal bovine serum (FBS; Gibco, Thermo Fisher Scientific, Waltham, MA, USA). Antibody titers ≥ 1.65 log₁₀ TCID₅₀ were considered protective and served as a benchmark for vaccine efficacy.

#### Determination of serological relationship (r₁-Value) and cross-neutralization.

On day 28 post-vaccination, sera from vaccinated animals were evaluated using the virus neutralization test (VNT) against FMDV serotypes SAT1 and SAT2 (Lib-12 and SAT2/EGY-2012) to determine the serological relationship (r₁-value). In addition, cross-neutralization was assessed by testing SAT1-vaccinated sera against the SAT2 strains to evaluate the extent of heterologous protection. Calculation and interpretation of r₁-values were performed following WOAH guidelines, providing a measure of both antigenic match and cross-reactive neutralizing capacity between the vaccine strain (SAT1) and other SAT2 viruses [21,23,24].

### Virus titration in calves’ tongue

The infectivity of the FMDV SAT1 strain, used for the challenge study, was determined by titration in the tongues of calves from Group 1. The procedure followed the protocols described by Dekker [25] and Abousenna [6], which specify methods for inoculation and assessment of viral infectivity in the target tissue. Virus titers were calculated using the Karber method [26] and expressed as log₁₀ Bovine Infective Dose (BID₅₀/mL), ensuring accurate and reliable determination of viral infectivity.

### Challenge Test in Vaccinated Calves

At 28 days following vaccination, calves assigned to the vaccinated group (Group 2), together with their corresponding unvaccinated positive control animals (Group 3), were relocated to a designated high-containment challenge unit within the animal facility. vaccinated calves and unvaccinated positive controls were challenged with the homologous SAT1 field isolate using a total challenge dose of 0.2 mL containing 10⁴ BID₅₀/mL. The inoculum was administered intradermolingually at two sites (0.1 mL per site) in accordance with WOAH-recommended challenge procedures and previously described protocols [21]. Protective efficacy was assessed seven days post-challenge by determining the proportion of vaccinated calves that remained clinically protected, defined as the absence of FMD signs. A protective level of ≥ 75% was considered indicative of effective vaccine performance.

Clinical evaluation was performed according to WOAH criteria [21] for FMD. Animals were examined daily for 7 days post-challenge for rectal temperature, appetite, lameness, salivation, and vesicular lesions in the oral cavity and feet. Clinical protection was defined as the absence of generalized vesicular lesions in at least one foot and/or oral mucosa. Animals presenting with vesicles in multiple sites were classified as clinically infected.

All animals subjected to the challenge received appropriate care and management according to the procedures outlined by Abousenna [6].

### Humane endpoints, clinical monitoring, and supportive care

Death was not a planned experimental endpoint in any animal group. All calves, including those used for tongue titration and challenge studies, were maintained under veterinary supervision and monitored daily for general health, feed and water intake, body temperature, activity, pain-related behaviors, salivation, oral lesions, lameness, and other clinical signs associated with foot-and-mouth disease. Animals showing abnormal signs were monitored more frequently as required.

Predefined humane endpoints included severe or progressive distress, persistent recumbency, inability to access feed or water, marked respiratory compromise, severe oral lesions interfering with feeding, severe lameness impairing normal movement, or any condition judged by the attending veterinarian to compromise animal welfare. Animals meeting these criteria would have been immediately removed from the study and humanely euthanized according to institutional animal-care procedures. No animals reached humane endpoints, and no unexpected mortality occurred during the study.

### Ethical approval for the animal experiments

This study was conducted in accordance with the ARRIVE guidelines and approved by the Agricultural Research Center Institutional Animal Care and Use Committee (ARC-IACUC; approval no. ARC-CLEVB-88–25). All animal procedures complied with institutional ethical standards and veterinary welfare requirements. Animals were clinically monitored throughout the study, and humane endpoints were established for all regulated-animal procedures. No animals required euthanasia during the experiment.

### Statistical analysis

Statistical analyses were conducted in accordance with the study objectives and the WOAH guidelines for evaluation of inactivated FMD vaccines. As vaccine performance under WOAH standards is determined by compliance with predefined immunological and protection thresholds, analyses were primarily descriptive and criterion-based.

#### Manufacturing consistency.

Viral titers (log₁₀ TCID₅₀/mL) and 146S antigen concentrations (µg/mL) from 16 independent production sub-batches were summarized as mean ± standard deviation (SD). Batch-to-batch variability was assessed descriptively to evaluate production uniformity relative to predefined acceptance criteria.

#### Immunogenicity assessment.

Virus neutralization titers were summarized as mean ± standard deviation (SD) and 95% confidence intervals (CI). Differences between pre-vaccination (Day 0) and post-vaccination (Day 28) neutralizing antibody titers were evaluated using the Wilcoxon signed-rank test because of the small sample size and paired study design. Statistical significance was defined as P < 0.05.

Protection following homologous challenge was expressed as a proportion with exact binomial 95% confidence intervals (Clopper–Pearson method).

Antigenic matching (r₁-value).

r₁-values were calculated as the ratio of heterologous to homologous neutralization titers. Antigenic relationships were interpreted using WOAH criteria (r₁ ≥ 0.3 indicating an ac-acceptable match). Interpretation was categorical and threshold-based.

#### Protective efficacy.

Protective efficacy was calculated as the percentage of vaccinated animals that did not develop generalized disease following challenge. Vaccine efficacy was considered satisfactory when ≥75% of animals were clinically protected, in accordance with WOAH standards.

All statistical analyses were performed using Python version 3.10.12 (Python Software Foundation, Wilmington, DE, USA) with the NumPy package. Figures were generated using Matplotlib (Matplotlib Development Team) within Python to ensure transparent, script-based, and reproducible data processing and visualization. Data are presented as mean ± SD or percentages, as appropriate

## Results

### In-process evaluation of the inactivated SAT1 monovalent FMD vaccine

#### In-process verification of SAT1 viral identity and quantification.

Real-time RT-PCR analysis confirmed the presence and genetic identity of FMDV SAT1 in all 16 in-process sub-batches prior to chemical inactivation. All amplification controls (APC and ANC) and extraction controls (PPC and PNC) performed within acceptable limits, verifying the reliability of the assay and the absence of contamination or inhibitory effects.

Ct values across sub-batches ranged from 11.0 to 12.8, with most values below 12.5. These low Ct values are consistent with high viral RNA loads and indicate successful replication during seed-virus propagation.

Viral titers were calculated using the established standard-curve regression equation: Titer=−0.2953 × Ct + 12.24, All sub-batches surpassed the minimum potency requirement of ≥7.0 log₁₀ TCID₅₀/mL, with titers ranging from 7.66 to 8.42 log₁₀ TCID₅₀/mL (Table 1). Sub-batches 3, and 15 exhibited the highest titers (≥8.4 log₁₀ TCID₅₀/mL), confirming strong virus propagation suitable for downstream vaccine formulation.

**Table 1:** Calculated viral titers of SAT1 sub-batches during in-process verification prior to chemical inactivation.

Sub-Batch	1	2	3	4	5	6	7	8	9	10	11	12	13	14	15	16
**Ct Value**	12.4	11.8	11.2	12.0	12.6	12.1	11.5	12.3	11.9	12.8	11.6	12.2	12.5	11.7	11.0	12.0
**Titer (log₁₀ TCID₅₀/mL)**	*7.87	8.03	8.42	7.97	7.72	7.94	8.09	7.90	8.00	7.66	8.07	7.92	7.84	8.04	8.42	7.97
**Mean ± SD**	**7**.98 ± 0.23 log₁₀TCID₅₀/mL															

*Calculated viral titers of SAT1 sub-batches prior to chemical inactivation. Ct values were converted to log₁₀ TCID₅₀/mL using the standard curve. All sub-batches exceeded the minimum acceptable titer of 7.0 log₁₀ TCID₅₀/mL, consistent with typical industrial harvest titers.

#### Identification of prepared inactivated SAT1 antigen using FMDV serotyping ELISA.

All 16 inactivated SAT1 antigen sub-batches were evaluated for serotype identity using FMDV Serotyping ELISA. Each sub-batch exhibited strong reactivity exclusively in SAT1-specific wells, with no detectable signal observed in wells corresponding to serotypes O, A, SAT2, or Asia 1.

#### Quantification of 146S antigen content in in-process SAT1 sub-batches.

The intact 146S virion particles were quantified in all 16 SAT1 in-process antigen sub-batches using sucrose density-gradient ultracentrifugation with UV absorbance integration. All sub-batches produced distinct 146S peaks, confirming preservation of structurally intact virions. Concentrations ranged from 14.0 to 17.7 µg/mL, with a mean of 15.8 ± 1.1 µg/mL, demonstrating minimal batch-to-batch variability. All sub-batches met internal manufacturing acceptance criteria for antigen content and were suitable for downstream formulation of the monovalent SAT1 vaccine as indicated in Table 2.

**Table 2:** 146S Antigen Content of SAT1 In-Process Sub-Batches.

Sub-Batch	*1	2	3	4	5	6	7	8	9	10	11	12	13	14	15	16
**146S (µg/mL)**	15.2	15.8	17.7	15.6	14.0	15.4	16.2	15.0	15.7	14.0	16.0	15.1	15.0	16.1	17.6	15.5
**Mean ± SD**	15.8 ± 1.1 µg/mL															

*All sub-batches produced structurally intact 146S virions, with concentrations ranging from 14.0 to 17.7 µg/mL (mean ± SD: 15.8 ± 1.1 µg/mL). These values represent the concentration of 146S antigen in the inactivated bulk harvest prior to formulation. The final vaccine dose was subsequently standardized during oil-adjuvant emulsification to contain approximately 7 µg of 146S antigen per dose.

### Final product evaluation of the inactivated SAT1 monovalent FMD vaccine

#### Vaccine safety.

All calves vaccinated with the SAT1 monovalent inactivated FMD vaccine remained clinically healthy throughout the observation period. No local reactions at the injection sites, such as swelling, redness, or induration, were detected in any animal. Daily body temperature measurements remained within the normal physiological range for all calves, with no evidence of pyrexia or other systemic responses. Overall, the vaccine was well-tolerated, demonstrating an excellent safety profile with no post-vaccination adverse effects observed in any of the animals.

#### Virus neutralization antibody responses following vaccination.

Pre-vaccination sera exhibited low neutralizing activity against the SAT1 vaccine strain, with a mean VNT titer of 0.169 ± 0.125 log_10_ TCID_50_ (95% CI: 0.064–0.273). At 28 days post-vaccination, calves immunized with the monovalent inactivated SAT1 FMDV vaccine developed strong homologous neutralizing antibody responses as measured by the virus neutralization test (VNT). The mean neutralizing antibody titer against the homologous SAT1 strain of 2.175 ± 0.266 log_10_ TCID_50_. The corresponding 95% CI was 1.95–2.40 log_10_ TCID_50_(Wilcoxon signed-rank test, W = 0, P = 0.0078), indicating a consistently strong antibody response among vaccinated animals. exceeding the WOAH-defined protective threshold of 1.65 log₁₀ TCID₅₀ (Table 3).

**Table 3:** Virus neutralization titers against homologous SAT1 and heterologous SAT2 vaccine strains.

Virus strain (tested antigen)	Vaccine status	Serotype/ lineage	Mean VNT titer (log₁₀ TCID₅₀) (Pre-vaccination)	Mean VNT titer (log₁₀ TCID₅₀) (28^th^ Day)
SAT1 vaccine strain	Homologous	SAT1 Topotype I	0.169	2.175
SAT2/VII (Lib-12)	Heterologous vaccine strain	SAT2/ VII/ Gharbia-12		0.26
SAT2/EGY-2012	Heterologous vaccine strain	SAT2/VII/ Lib-12		0.30

*Protective threshold defined as ≥1.65 log₁₀ TCID₅₀ according to WOAH recommendations.

Table 3. Virus neutralization antibody titers in calves vaccinated with a monovalent inactivated SAT1 FMD vaccine at 28 days post-vaccination, measured against homologous SAT1 and heterologous SAT2 vaccine strains.

In contrast, sera from SAT1-vaccinated calves demonstrated low heterologous neutralizing activity against the tested SAT2 strains. Mean VNT titers against SAT2/VII (Lib-12) and SAT2/EGY-2012 (SAT2 GH) were 0.26 and 0.3 log₁₀ TCID₅₀, respectively, remaining well below the protective cutoff (Table 3).

#### Antigenic relationship and cross-neutralization analysis.

Serological relationship analysis based on r₁-values revealed limited antigenic similarity between the SAT1 vaccine strain and the evaluated SAT2 field strains. The calculated r₁-values were 0.12 for SAT2/EGY-2012 and 0.14 for SAT2/VII (Lib-12) (Table 4).

**Table 4:** Antigenic relationship (r₁-values) between SAT1 vaccine strain and SAT2 vaccine strains.

Reference vaccine strain	Heterologous vaccine strain	Serotype/ lineage	r₁-value	Antigenic relationship interpretation*
SAT1 (monovalent vaccine)	SAT2/EGY-2012	SAT2/VII/ Lib-12	0.12	Poor antigenic match
SAT1 (monovalent vaccine)	SAT2/VII (Lib-12)	SAT2/ VII/ Gharbia-12	0.14	Poor antigenic match

*r₁-values < 0.3 indicate insufficient antigenic similarity and a low likelihood of meaningful cross-protection, according to WOAH guidelines.

Table 4. r₁-values indicating the antigenic relationship between the SAT1 monovalent vaccine strain and heterologous SAT2 vaccine strains.

Both values fell below the WOAH-recommended threshold of 0.3, indicating poor antigenic matching and a low likelihood of meaningful cross-protection. Based on these findings, in vivo challenge experiments against heterologous SAT2 strains were not pursued.

#### Interpretation and decision on heterologous challenge.

Based on the r₁-values (<0.3) and the observed heterologous VNT titers (<1.65 log₁₀ TCID₅₀), heterologous challenge studies against SAT2 were not conducted.

#### Challenge virus titration.

The homologous SAT1 challenge virus was titrated in vivo in calves (Group 1). Viral titer was calculated using the Kärber method and expressed as log₁₀ BID₅₀/mL. The challenge virus titer was 7.0 log₁₀ BID₅₀/mL.

#### Protective efficacy following homologous challenge.

All vaccinated calves (8/8) remained clinically protected following challenge with the homologous SAT1 strain, resulting in a protection rate of 100% (95% CI: 63.1–100%) (Table 5). No generalized foot-and-mouth disease lesions were observed during the post-challenge observation period.

**Table 5:** Protective efficacy of SAT1 monovalent inactivated FMD vaccine in calves following homologous challenge.

Group	Group ID	Number of animals	Clinical protection (no lesions)	Protection rate (%)
Vaccinated (SAT1)	2	8	8	100
Unvaccinated control	3	2	0	0

Protection level: All vaccinated calves (Group 2) were clinically protected against the homologous SAT1 challenge, corresponding to a 100% protection rate, which exceeds the WOAH minimum efficacy criterion (≥75%) for FMD vaccines in cattle. Unvaccinated controls (Group 3) developed characteristic FMD lesions, confirming the infectivity and adequacy of the challenge virus.

In contrast, all unvaccinated control calves (2/2) developed generalized foot-and-mouth disease characterized by vesicular lesions in the tongue, oral cavity, and feet, accompanied by pyrexia and lameness, confirming the virulence of the challenge virus and the appropriateness of the experimental infection model. The observed protection level in vaccinated animals exceeded the minimum efficacy criterion (≥75%) recommended by WOAH for FMD vaccines in cattle.

## Discussion

This study provides a comprehensive evaluation of an emergency monovalent SAT1 foot-and-mouth disease (FMD) vaccine developed in response to Egypt’s first documented SAT1 incursion during July–August 2025. Although homologous protection against SAT1 is an expected feature of a SAT1-based vaccine, the novelty of this study lies in its integrated evaluation design rather than in demonstrating protection alone. The present work combines upstream production controls (RT-qPCR viral verification and batch-to-batch titration), structural antigen assessment (146S particle quantification), antigenic identity confirmation, and downstream immunological and protection endpoints within a single experimental framework. This multi-level approach enables direct correlation between vaccine production quality and biological performance, which is not routinely addressed in conventional FMD vaccine studies that often evaluate these components separately.

Despite the accelerated production timeline, the vaccine demonstrated high manufacturing consistency and antigen quality across all assessed parameters. Real-time RT-PCR analysis of 16 in-process sub-batches revealed uniformly high viral titers (7.98 ± 0.23 log₁₀ TCID₅₀/mL) with minimal variability, reflecting the robustness and reproducibility of the virus propagation process. Such consistency is critical for emergency vaccine production, where deviations in antigen yield or quality could compromise field efficacy.

Preservation of antigenic integrity following chemical inactivation was confirmed by serotype-specific ELISA, indicating that the inactivation protocol effectively abolished infectivity without compromising immunogenic epitopes. The relatively high mean 146S antigen concentration observed in the present study (15.8 ± 1.1 µg/mL) reflects the antigen content of the concentrated inactivated SAT1 bulk harvest prior to vaccine formulation rather than the final antigen payload per administered dose. Quantification was performed during the in-process quality-control stage to verify manufacturing consistency and ensure adequate antigen recovery from BHK-21 cell cultures. During vaccine formulation, the concentrated antigen was emulsified with the oil adjuvant and adjusted to the target antigen concentration required for the final field dose. Consequently, the final vaccine was standardized to contain approximately 7 µg of 146S antigen per dose, consistent with accepted potency requirements for inactivated FMD vaccines. Therefore, the value of 15.8 µg/mL should be interpreted as an indicator of efficient antigen production and recovery rather than the antigen content administered to vaccinated animals [20,27]. The narrow variability observed across production batches aligns with established evidence linking consistent 146S content to reliable vaccine potency [27].

When benchmarked against international SAT1 vaccine production data, the Egyptian vaccine compares favorably. Similar antigen quantification strategies have been reported for multivalent vaccines produced in South Africa, underscoring the universal importance of 146S measurement as a core quality control parameter [28]. Collectively, these findings demonstrate that VSVRI possesses the technical capacity to manufacture SAT1 vaccines that meet international quality expectations, even under emergency conditions.

Immunogenicity and challenge studies confirmed the strong homologous efficacy of the vaccine. Neutralizing antibody titers reached a mean of 2.175 log₁₀ TCID₅₀ by 28 days post-vaccination, clearly exceeding the WOAH-defined protective threshold of 1.65 log₁₀ TCID₅₀. This serological response translated into complete clinical protection (100%, 8/8 calves) following homologous challenge, surpassing the minimum WOAH requirement of ≥75% protection. These results are consistent with reports from East and Southern Africa, where SAT1 vaccine-induced titers typically range between 1.8 and 2.3 log₁₀ TCID₅₀ and protection rates vary from 83% to 100% [28,29].

The significance of achieving full protection is amplified by the epidemiological context. SAT1 topotype I had not previously circulated in Egypt, and the livestock population lacked pre-existing immunity [15,16]. Animals vaccinated with conventional trivalent vaccines (O, A, SAT2) were therefore immunologically unprotected during the initial outbreak [16,17]. The demonstrated efficacy of the monovalent SAT1 vaccine confirms its suitability for closing this immunity gap and controlling outbreaks in a fully susceptible population.

A key objective of this study was to investigate field observations suggesting milder clinical disease in some cattle vaccinated with SAT2-containing polyvalent vaccines during the SAT1 outbreak. These observations raised the hypothesis of possible partial cross-reactive immunity between SAT1 and SAT2. However, systematic antigenic matching and cross-neutralization analyses demonstrated r₁-values of only 0.12–0.14 between SAT1 vaccine sera and representative SAT2 strains, well below the WOAH threshold of 0.3 for meaningful antigenic relatedness. These results clearly indicate the absence of effective cross-protection and confirm the strictly serotype-specific nature of FMDV immunity.

Comparable findings have been reported in multiple international studies, where r₁-values between SAT1 and SAT2 consistently fall below 0.2, with no evidence of protective cross-neutralization [28–31]. The current data therefore validate the strategic decision to develop a dedicated SAT1 vaccine rather than relying on indirect protection from existing SAT2 components. The discrepancy between laboratory findings and anecdotal field observations may reflect non-neutralizing antibody effects, innate immune priming, mixed infections, or misclassification of clinical cases, emphasizing the necessity of laboratory-confirmed diagnostics during outbreak investigations. These findings are consistent with recent vaccine-matching and post-vaccination assessment studies conducted in endemic settings, which demonstrate that low r₁-values are predictive of limited heterologous protection and inform rational vaccine deployment strategies [32].

The poor antigenic relationship between SAT1 and SAT2 observed here is consistent with the broader antigenic diversity of FMDV and mirrors previous findings in Egypt, where newly emerged lineages of serotypes A and SAT2 showed limited vaccine matching, with r₁-values frequently below 0.3 [6,11,12]. This antigenic divergence is likely attributable to structural and amino-acid differences within key capsid regions of SAT serotypes that govern receptor binding and neutralizing antibody recognition, as previously demonstrated for SAT viruses [33]. These data reinforce the principle that both intra- and inter-serotype antigenic variation necessitate continuous surveillance and periodic vaccine updates.

International experience further contextualizes the Egyptian findings. While SAT1 vaccines in East Africa and Southern Africa often achieve high short-term efficacy, several studies have highlighted variability in performance and a tendency for SAT1 antibody titers to decline more rapidly than those against serotypes O and A, sometimes falling below protective levels within 4–6 months [30,34]. South African data suggest somewhat longer durability, with protective titers maintained for up to 6–8 months, potentially reflecting differences in strain selection and manufacturing optimization [28]. These observations indicate that SAT1 control may require tailored vaccination schedules or higher antigen payloads to ensure sustained immunity.

Phylogenetic evidence linking the Egyptian SAT1 isolate to North-West Zimbabwe lineages within topotype I highlights the transboundary nature of FMDV spread and parallels previous incursions of exotic lineages into Egypt, including A EURO-SA and SAT2 Lib-12 [10,12,14,15]. Such patterns underscore Egypt’s vulnerability to long-distance viral introductions and the necessity for regional coordination, harmonized surveillance, and rapid vaccine adaptation.

From a policy perspective, the absence of cross-protection between SAT1 and existing vaccine components necessitates immediate adjustments to vaccination strategies. Reliance on trivalent vaccines alone leaves a substantial immunity gap, as demonstrated during the initial outbreak [16,17]. While monovalent SAT1 vaccination is appropriate as an emergency response, long-term control will likely depend on integrating SAT1 into routine polyvalent formulations. International experience with quadrivalent vaccines (O, A, SAT1, SAT2) provides valuable guidance but also highlights technical challenges related to antigen balance, stability, and manufacturing complexity [29,32].

Egypt’s prior experience updating polyvalent vaccines to include newly emerged lineages [11,12,19] provides a practical framework for SAT1 integration. However, careful optimization will be required to avoid antigenic competition and ensure balanced immune responses. Decisions regarding routine inclusion of SAT1 should be guided by ongoing surveillance data on viral persistence and geographic spread.

The successful development and evaluation of this emergency SAT1 vaccine demonstrate Egypt’s capacity for rapid, high-quality vaccine production. The quality control framework employed—combining RT-PCR quantification, serotype-specific ELISA, and 146S particle measurement—aligns with international best practices [28,35,36] and provides strong support for regulatory approval and international confidence in the product.

Statistical analysis further supported the immunogenicity of the emergency SAT1 vaccine. Neutralizing antibody titers increased significantly between pre-vaccination and post-vaccination sampling (P = 0.0078), with all vaccinated animals developing antibody levels exceeding the accepted protective threshold. In addition, the challenge study demonstrated complete clinical protection, corresponding to an exact 95% confidence interval of 63.1–100%. Although the confidence interval remains relatively wide because of the limited sample size, these findings provide strong evidence of homologous protective efficacy under controlled experimental conditions.

The emergence of SAT1 in Egypt reflects the broader global epidemiological patterns of FMD characterized by transboundary spread and continual viral evolution, underscoring the need for sustained surveillance and adaptive vaccination strategies [37]. Emergent vaccine research continues to inform policy frameworks for FMD control, emphasizing both targeted serotype responses and integrated antigenic matching approaches [18].

### Limitations

This study has several limitations that should be acknowledged. The number of animals included in the immunogenicity and challenge experiments was limited and followed the minimum sample size commonly used in preliminary FMD vaccine evaluation studies. While adequate to generate initial immunogenicity and protection data, this design limits the assessment of inter-individual variability and reduces the statistical power for precise potency estimation.

Immunological assessment was restricted to neutralizing antibody responses measured at 28 days post-vaccination. Consequently, long-term antibody persistence, anamnestic responses, and cell-mediated immunity were not evaluated, although these may contribute to sustained protection against FMD.

In addition, sterile immunity following challenge was not investigated, as post-challenge viral load quantification (e.g., RT-qPCR detection of viral RNA in blood or oropharyngeal samples) was not performed. Therefore, viral clearance kinetics and potential subclinical or transient infection could not be assessed. The primary endpoint was limited to clinical protection, consistent with WOAH-recommended criteria for emergency FMD vaccine evaluation.

Assessment of heterologous protection relied on in vitro virus neutralization and r₁-value analysis using SAT2 reference strains rather than contemporary field isolates. The low r₁-values observed indicated minimal antigenic cross-reactivity, supporting the decision not to perform heterologous challenge experiments, in accordance with WOAH guidance.

Finally, the findings are specific to a single emergency-produced monovalent SAT1 vaccine batch and should not be directly extrapolated to other strains, production platforms, or multivalent formulations without further validation. Although the integrated evaluation framework combines antigen quality, batch consistency, and antigenic matching in line with WOAH recommendations, this multi-parameter design may limit detailed inference on any single component when interpreted in isolation. Nevertheless, it reflects a pragmatic approach for emergency vaccine assessment where complementary in-process and functional data are required to support rapid decision-making and vaccine refinement.

## Conclusion

This study provides an integrated, WOAH-aligned preliminary evaluation of an emergency monovalent SAT1 vaccine, generating early evidence to support vaccine deployment and refinement during an unprecedented SAT1 outbreak in Egypt. The vaccine elicited robust homologous neutralizing antibody responses and conferred complete clinical protection against SAT1 challenge, effectively addressing the immunity gap created by the recent emergence of SAT1 in a previously naïve livestock population.

Systematic evaluation of antigenic relationships between SAT1 and SAT2 confirmed the absence of meaningful cross-reactive immunity, with r₁-values of 0.12–0.14. These findings validate the serotype-specific nature of FMD immunity and underscore the necessity for dedicated SAT1 vaccination, despite anecdotal field observations suggesting possible modulation of disease severity in SAT2-vaccinated animals.

The results support the immediate use of the monovalent SAT1 vaccine as an effective emergency control measure and highlight the need to consider SAT1 integration into routine polyvalent vaccination programs for sustained protection. More broadly, this work demonstrates the importance of rapid vaccine adaptation, continuous antigenic surveil-lance, and evidence-based policy decisions in responding to the evolving epidemiology of FMD, with implications extending beyond Egypt to other regions at risk of transboundary viral incursions.

**Institutional Review Board Statement:** The current study followed the Animal Research: Reporting of In-Vivo Experiments (ARRIVE) guidelines. All procedures involving animal use strictly adhered to the guidelines established by the ARC-IACUC. Ethical approval for this study was obtained from the Committee (ARC-IACUC) approval No (ARC-CLEVB-88–25). The manuscript is considered com-pliant with bioethical standards in good faith. No anesthesia or euthanasia protocols were employed for the animals involved in this study, as all animal-dependent methodological procedures were categorized as either no or low-pain procedures that can be ethically performed on a conscious and living animal.

## Supporting information

S1 Fig FMD SAT1 qPCR standard curve with linear equation. FMD SAT1 qPCR standard curve used for in-process verification of viral identity and quantification. Ct values obtained from 16 independent sub-batches were converted to viral RNA copy numbers using the linear regression equation (y = −0.2953x + 12.24; R² = 0.9967). This standard curve demonstrates the dynamic range of the assay (10¹–10⁷ copies/µL) and allows accurate calculation of viral titers for quality control of vaccine sub-batches.
(TIF)

## Abbreviations

AHRI: Animal Health Research Institute
ANC: Amplification Negative Control
APC: Amplification Positive Control
ARC: Agricultural Research Center
ARRIVE: Animal Research: Reporting of In Vivo Experiments
BEI: Binary Ethyleneimine
BHK-21: Baby Hamster Kidney cell line
BID₅₀: Bovine Infective Dose 50%
CI: Confidence Interval
CLEVB: Central Laboratory for Evaluation of Veterinary Biologics
Ct: Cycle threshold
DMEM: Dulbecco’s Modified Eagle Medium
ELISA: Enzyme-Linked Immunosorbent Assay
EURO-SA: Europe–South America lineage
FAM: 6-Carboxyfluorescein
FBS: Fetal Bovine Serum
FMD: Foot-and-Mouth Disease
FMDV: Foot-and-Mouth Disease Virus
GH: Gharbia
GMP: Good Manufacturing Practice
IACUC: Institutional Animal Care and Use Committee
ISA: Montanide Incomplete Seppic Adjuvant (ISA 206)
IZSLER: Istituto Zooprofilattico Sperimentale della Lombardia e dell’Emilia Romagna
kb: Kilobase
Lib-12: Libya 2012 lineage
ME-SA: Middle East–South Asia
PEG: Polyethylene Glycol
PNC: Purification Negative Control
PPC: Purification Positive Control
qPCR: Quantitative Polymerase Chain Reaction
r₁-value: Serological relationship coefficient
RNA: Ribonucleic Acid
RT-PCR: Reverse Transcription Polymerase Chain Reaction
RT-qPCR: Reverse Transcription Quantitative PCR
SAT: South African Territories
SD: Standard Deviation
TCID₅₀: Tissue Culture Infective Dose 50%
UV: Ultraviolet
VNT: Virus Neutralization Test
VP1: Viral Protein 1
VSVRI: Veterinary Serum and Vaccine Research Institute
WOAH: World Organisation for Animal Health
WRLFMD: World Reference Laboratory for Foot-and-Mouth Disease
